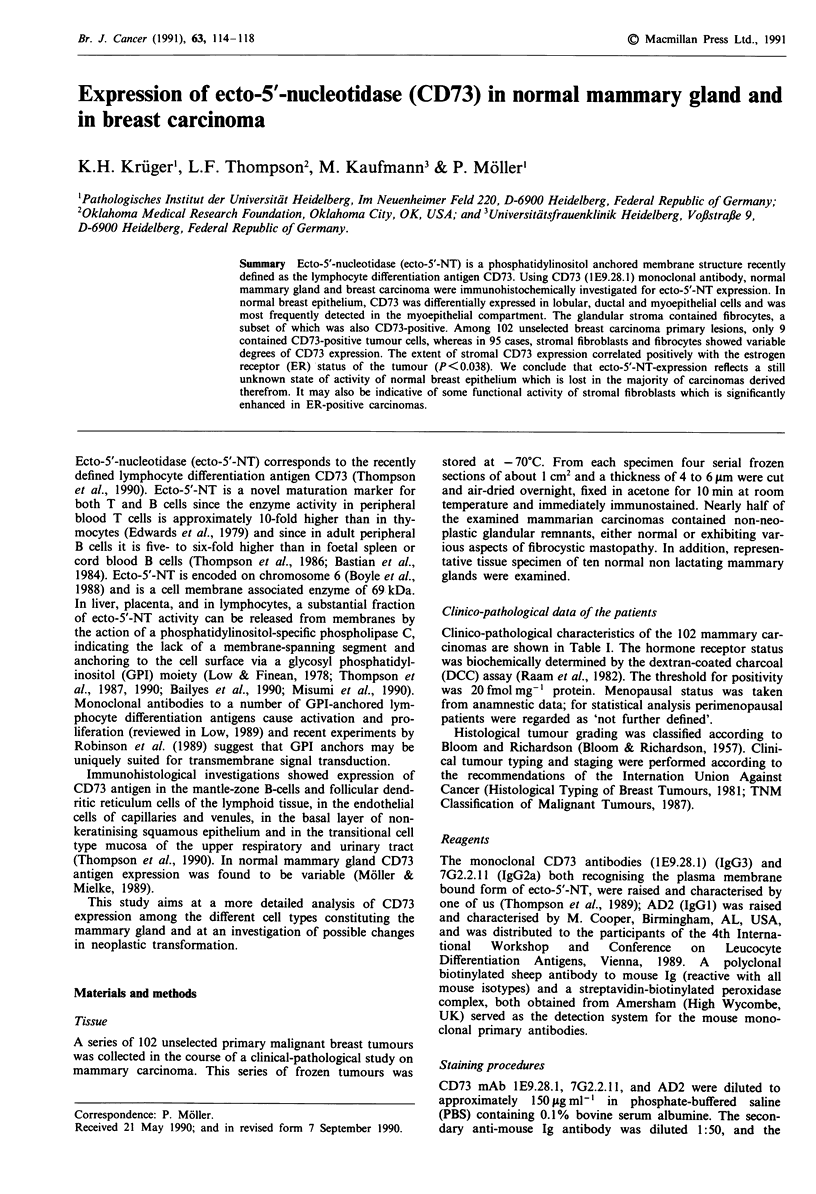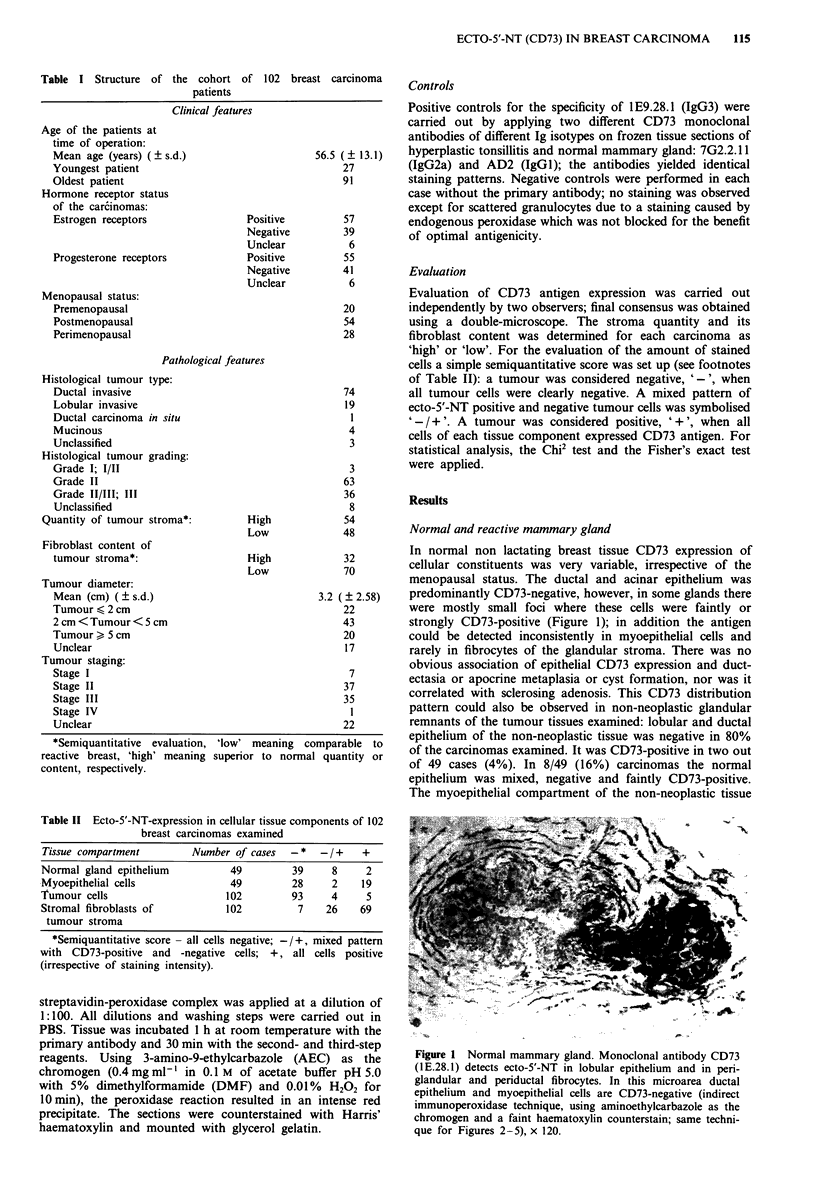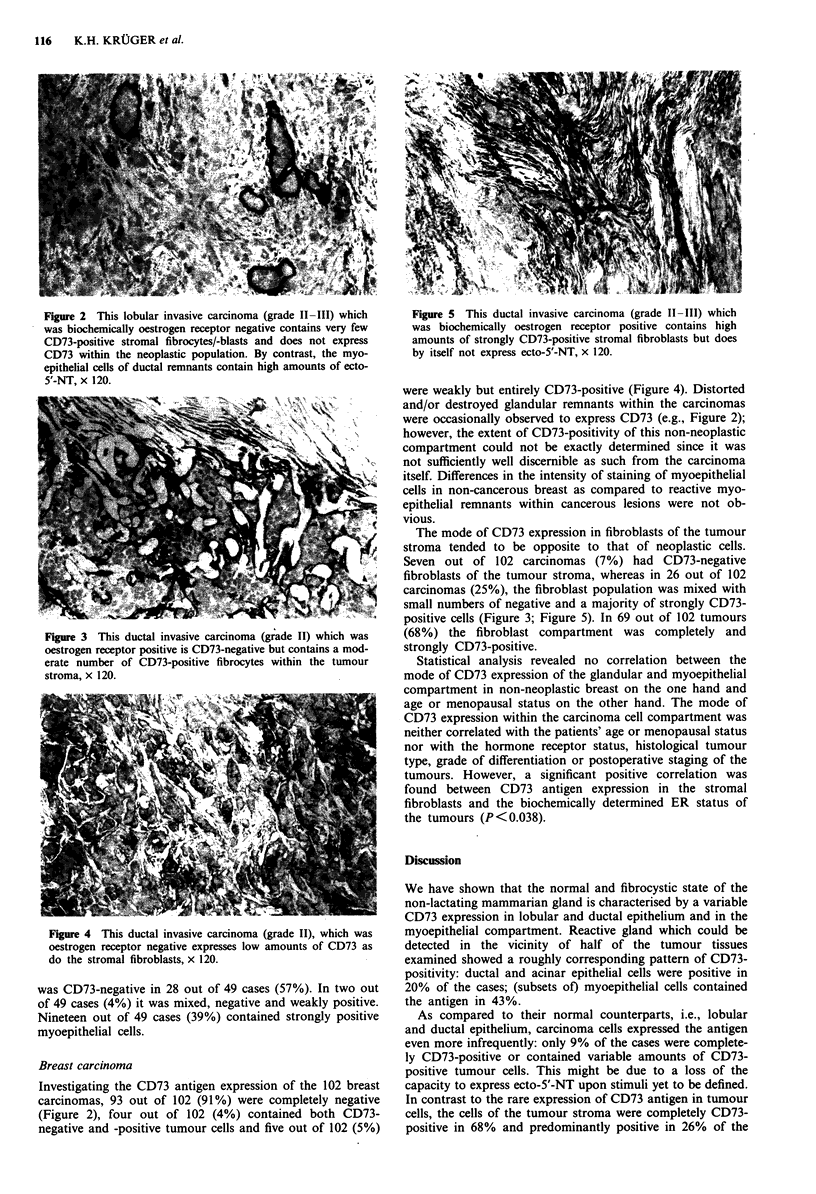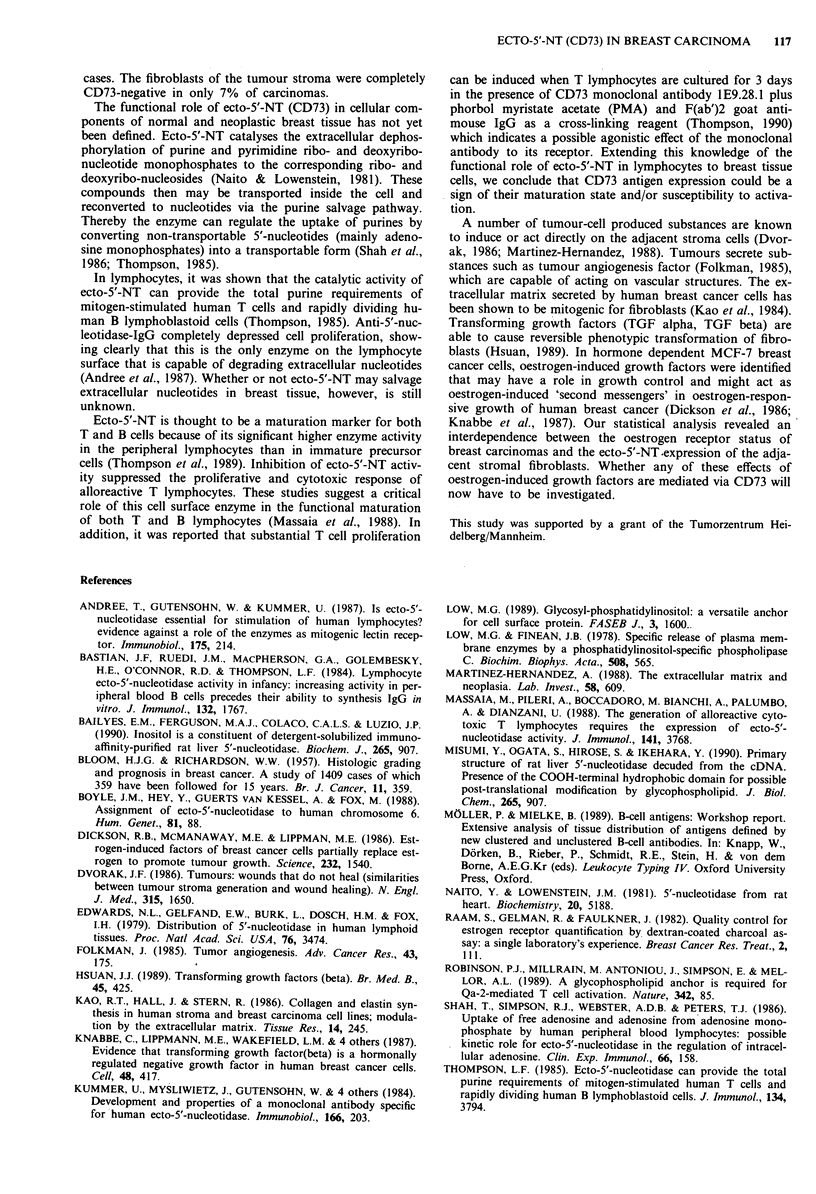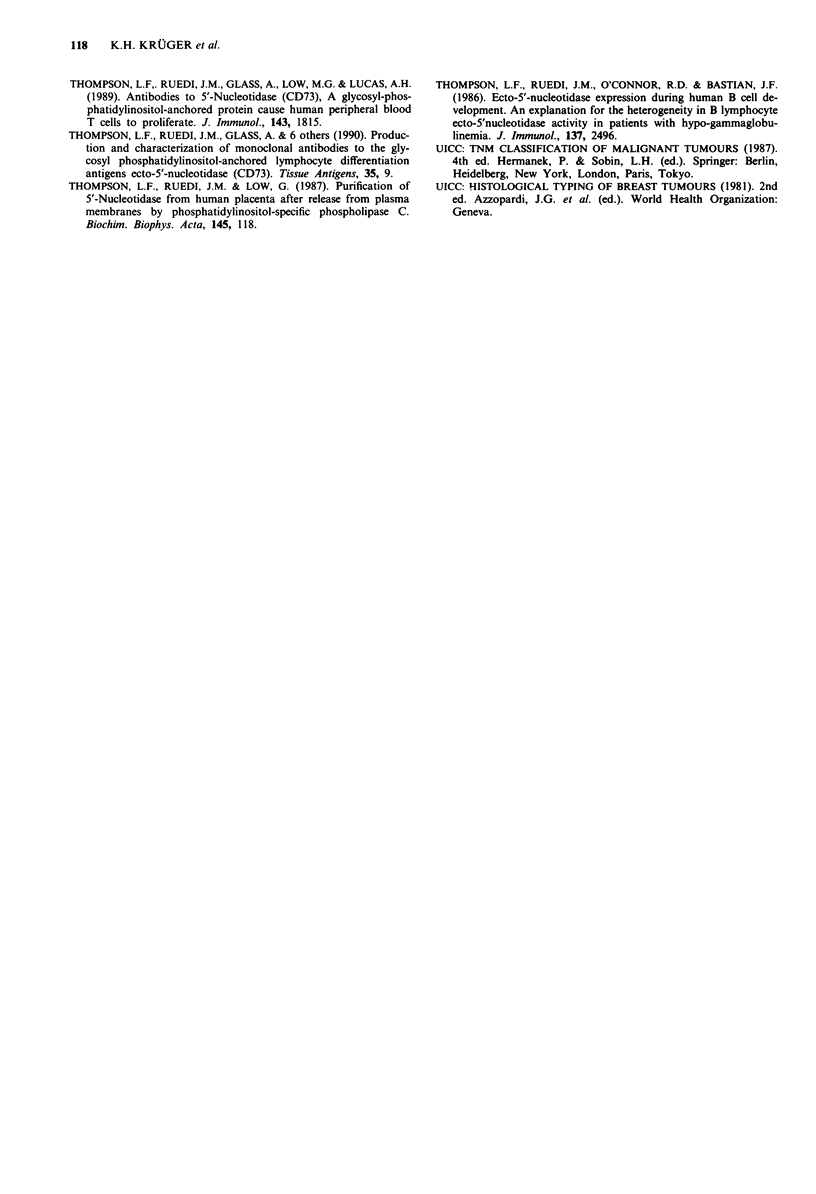# Expression of ecto-5'-nucleotidase (CD73) in normal mammary gland and in breast carcinoma.

**DOI:** 10.1038/bjc.1991.23

**Published:** 1991-01

**Authors:** K. H. Krüger, L. F. Thompson, M. Kaufmann, P. Möller

**Affiliations:** Pathologisches Institut, Universität Heidelberg, Federal Republic of Germany.

## Abstract

**Images:**


Br. J. Cancer (1991), 63, 114–118 © Macmillan Press Ltd., 1991

### Expression of ecto-5′-nucleotidase (CD73) in normal mammary gland and in breast carcinoma

K.H. Krüger[1], L.F. Thompson[2], M. Kaufmann[3] & P. Möller[1]

[1]*Pathologisches Institut der Universität Heidelberg, Im Neuenheimer Feld 220, D-6900 Heidelberg, Federal Republic of Germany;* [2]*Oklahoma Medical Research Foundation, Oklahoma City, OK, USA; and* [3]*Universitätsfrauenklinik Heidelberg, Voßstraße 9, D-6900 Heidelberg, Federal Republic of Germany.*

**Summary** Ecto-5′-nucleotidase (ecto-5′-NT) is a phosphatidylinositol anchored membrane structure recently defined as the lymphocyte differentiation antigen CD73. Using CD73 (1E9.28.1) monoclonal antibody, normal mammary gland and breast carcinoma were immunohistochemically investigated for ecto-5′-NT expression. In normal breast epithelium, CD73 was differentially expressed in lobular, ductal and myoepithelial cells and was most frequently detected in the myoepithelial compartment. The glandular stroma contained fibrocytes, a subset of which was also CD73-positive. Among 102 unselected breast carcinoma primary lesions, only 9 contained CD73-positive tumour cells, whereas in 95 cases, stromal fibroblasts and fibrocytes showed variable degrees of CD73 expression. The extent of stromal CD73 expression correlated positively with the estrogen receptor (ER) status of the tumour ($P < 0.038$). We conclude that ecto-5′-NT-expression reflects a still unknown state of activity of normal breast epithelium which is lost in the majority of carcinomas derived therefrom. It may also be indicative of some functional activity of stromal fibroblasts which is significantly enhanced in ER-positive carcinomas.

Ecto-5′-nucleotidase (ecto-5′-NT) corresponds to the recently defined lymphocyte differentiation antigen CD73 (Thompson *et al.*, 1990). Ecto-5′-NT is a novel maturation marker for both T and B cells since the enzyme activity in peripheral blood T cells is approximately 10-fold higher than in thymocytes (Edwards *et al.*, 1979) and since in adult peripheral B cells it is five- to six-fold higher than in foetal spleen or cord blood B cells (Thompson *et al.*, 1986; Bastian *et al.*, 1984). Ecto-5′-NT is encoded on chromosome 6 (Boyle *et al.*, 1988) and is a cell membrane associated enzyme of 69 kDa. In liver, placenta, and in lymphocytes, a substantial fraction of ecto-5′-NT activity can be released from membranes by the action of a phosphatidylinositol-specific phospholipase C, indicating the lack of a membrane-spanning segment and anchoring to the cell surface via a glycosyl phosphatidylinositol (GPI) moiety (Low & Finean, 1978; Thompson *et al.*, 1987, 1990; Bailyes *et al.*, 1990; Misumi *et al.*, 1990). Monoclonal antibodies to a number of GPI-anchored lymphocyte differentiation antigens cause activation and proliferation (reviewed in Low, 1989) and recent experiments by Robinson *et al.* (1989) suggest that GPI anchors may be uniquely suited for transmembrane signal transduction.

Immunohistological investigations showed expression of CD73 antigen in the mantle-zone B-cells and follicular dendritic reticulum cells of the lymphoid tissue, in the endothelial cells of capillaries and venules, in the basal layer of non-keratinising squamous epithelium and in the transitional cell type mucosa of the upper respiratory and urinary tract (Thompson *et al.*, 1990). In normal mammary gland CD73 antigen expression was found to be variable (Möller & Mielke, 1989).

This study aims at a more detailed analysis of CD73 expression among the different cell types constituting the mammary gland and at an investigation of possible changes in neoplastic transformation.

### Materials and methods

### *Tissue*

A series of 102 unselected primary malignant breast tumours was collected in the course of a clinical-pathological study on mammary carcinoma. This series of frozen tumours was stored at −70°C. From each specimen four serial frozen sections of about 1 $cm^2$ and a thickness of 4 to 6 µm were cut and air-dried overnight, fixed in acetone for 10 min at room temperature and immediately immunostained. Nearly half of the examined mammarian carcinomas contained non-neoplastic glandular remnants, either normal or exhibiting various aspects of fibrocystic mastopathy. In addition, representative tissue specimen of ten normal non lactating mammary glands were examined.

### *Clinico-pathological data of the patients*

Clinico-pathological characteristics of the 102 mammary carcinomas are shown in Table I. The hormone receptor status was biochemically determined by the dextran-coated charcoal (DCC) assay (Raam *et al.*, 1982). The threshold for positivity was 20 fmol $mg^{-1}$ protein. Menopausal status was taken from anamnestic data; for statistical analysis perimenopausal patients were regarded as 'not further defined'.

Histological tumour grading was classified according to Bloom and Richardson (Bloom & Richardson, 1957). Clinical tumour typing and staging were performed according to the recommendations of the Internation Union Against Cancer (Histological Typing of Breast Tumours, 1981; TNM Classification of Malignant Tumours, 1987).

### *Reagents*

The monoclonal CD73 antibodies (1E9.28.1) (IgG3) and 7G2.2.11 (IgG2a) both recognising the plasma membrane bound form of ecto-5′-NT, were raised and characterised by one of us (Thompson *et al.*, 1989); AD2 (IgG1) was raised and characterised by M. Cooper, Birmingham, AL, USA, and was distributed to the participants of the 4th International Workshop and Conference on Leucocyte Differentiation Antigens, Vienna, 1989. A polyclonal biotinylated sheep antibody to mouse Ig (reactive with all mouse isotypes) and a streptavidin-biotinylated peroxidase complex, both obtained from Amersham (High Wycombe, UK) served as the detection system for the mouse monoclonal primary antibodies.

### *Staining procedures*

CD73 mAb 1E9.28.1, 7G2.2.11, and AD2 were diluted to approximately 150 µg $ml^{-1}$ in phosphate-buffered saline (PBS) containing 0.1% bovine serum albumine. The secondary anti-mouse Ig antibody was diluted 1:50, and the

Correspondence: P. Möller.
Received 21 May 1990; and in revised form 7 September 1990.

**Table I** Structure of the cohort of 102 breast carcinoma patients

| | | |
|---|---|---|
| *Clinical features* | | |
| Age of the patients at time of operation: | | |
| Mean age (years) (± s.d.) | | 56.5 (± 13.1) |
| Youngest patient | | 27 |
| Oldest patient | | 91 |
| Hormone receptor status of the carcinomas: | | |
| Estrogen receptors | Positive | 57 |
| | Negative | 39 |
| | Unclear | 6 |
| Progesterone receptors | Positive | 55 |
| | Negative | 41 |
| | Unclear | 6 |
| Menopausal status: | | |
| Premenopausal | | 20 |
| Postmenopausal | | 54 |
| Perimenopausal | | 28 |
| *Pathological features* | | |
| Histological tumour type: | | |
| Ductal invasive | | 74 |
| Lobular invasive | | 19 |
| Ductal carcinoma *in situ* | | 1 |
| Mucinous | | 4 |
| Unclassified | | 3 |
| Histological tumour grading: | | |
| Grade I; I/II | | 3 |
| Grade II | | 63 |
| Grade II/III; III | | 36 |
| Unclassified | | 8 |
| Quantity of tumour stroma*: | High | 54 |
| | Low | 48 |
| Fibroblast content of tumour stroma*: | High | 32 |
| | Low | 70 |
| Tumour diameter: | | |
| Mean (cm) (± s.d.) | | 3.2 (± 2.58) |
| Tumour ⩽ 2 cm | | 22 |
| 2 cm < Tumour < 5 cm | | 43 |
| Tumour ⩾ 5 cm | | 20 |
| Unclear | | 17 |
| Tumour staging: | | |
| Stage I | | 7 |
| Stage II | | 37 |
| Stage III | | 35 |
| Stage IV | | 1 |
| Unclear | | 22 |

*Semiquantitative evaluation, 'low' meaning comparable to reactive breast, 'high' meaning superior to normal quantity or content, respectively.

**Table II** Ecto-5′-NT-expression in cellular tissue components of 102 breast carcinomas examined

| *Tissue compartment* | *Number of cases* | −* | −/+ | + |
|---|---|---|---|---|
| Normal gland epithelium | 49 | 39 | 8 | 2 |
| Myoepithelial cells | 49 | 28 | 2 | 19 |
| Tumour cells | 102 | 93 | 4 | 5 |
| Stromal fibroblasts of tumour stroma | 102 | 7 | 26 | 69 |

*Semiquantitative score − all cells negative; −/+, mixed pattern with CD73-positive and -negative cells; +, all cells positive (irrespective of staining intensity).

streptavidin-peroxidase complex was applied at a dilution of 1:100. All dilutions and washing steps were carried out in PBS. Tissue was incubated 1 h at room temperature with the primary antibody and 30 min with the second- and third-step reagents. Using 3-amino-9-ethylcarbazole (AEC) as the chromogen (0.4 mg ml$^{-1}$ in 0.1 M of acetate buffer pH 5.0 with 5% dimethylformamide (DMF) and 0.01% $H_2O_2$ for 10 min), the peroxidase reaction resulted in an intense red precipitate. The sections were counterstained with Harris' haematoxylin and mounted with glycerol gelatin.

### *Controls*

Positive controls for the specificity of 1E9.28.1 (IgG3) were carried out by applying two different CD73 monoclonal antibodies of different Ig isotypes on frozen tissue sections of hyperplastic tonsillitis and normal mammary gland: 7G2.2.11 (IgG2a) and AD2 (IgG1); the antibodies yielded identical staining patterns. Negative controls were performed in each case without the primary antibody; no staining was observed except for scattered granulocytes due to a staining caused by endogenous peroxidase which was not blocked for the benefit of optimal antigenicity.

### *Evaluation*

Evaluation of CD73 antigen expression was carried out independently by two observers; final consensus was obtained using a double-microscope. The stroma quantity and its fibroblast content was determined for each carcinoma as 'high' or 'low'. For the evaluation of the amount of stained cells a simple semiquantitative score was set up (see footnotes of Table II): a tumour was considered negative, '−', when all tumour cells were clearly negative. A mixed pattern of ecto-5′-NT positive and negative tumour cells was symbolised '−/+'. A tumour was considered positive, '+', when all cells of each tissue component expressed CD73 antigen. For statistical analysis, the Chi$^2$ test and the Fisher's exact test were applied.

### Results

### *Normal and reactive mammary gland*

In normal non lactating breast tissue CD73 expression of cellular constituents was very variable, irrespective of the menopausal status. The ductal and acinar epithelium was predominantly CD73-negative, however, in some glands there were mostly small foci where these cells were faintly or strongly CD73-positive (Figure 1); in addition the antigen could be detected inconsistently in myoepithelial cells and rarely in fibrocytes of the glandular stroma. There was no obvious association of epithelial CD73 expression and duct-ectasia or apocrine metaplasia or cyst formation, nor was it correlated with sclerosing adenosis. This CD73 distribution pattern could also be observed in non-neoplastic glandular remnants of the tumour tissues examined: lobular and ductal epithelium of the non-neoplastic tissue was negative in 80% of the carcinomas examined. It was CD73-positive in two out of 49 cases (4%). In 8/49 (16%) carcinomas the normal epithelium was mixed, negative and faintly CD73-positive. The myoepithelial compartment of the non-neoplastic tissue

**Figure 1** Normal mammary gland. Monoclonal antibody CD73 (1E.28.1) detects ecto-5′-NT in lobular epithelium and in periglandular and periductal fibrocytes. In this microarea ductal epithelium and myoepithelial cells are CD73-negative (indirect immunoperoxidase technique, using aminoethylcarbazole as the chromogen and a faint haematoxylin counterstain; same technique for Figures 2–5), × 120.

**Figure 2** This lobular invasive carcinoma (grade II–III) which was biochemically oestrogen receptor negative contains very few CD73-positive stromal fibrocytes/-blasts and does not express CD73 within the neoplastic population. By contrast, the myoepithelial cells of ductal remnants contain high amounts of ecto-5′-NT, × 120.

**Figure 3** This ductal invasive carcinoma (grade II) which was oestrogen receptor positive is CD73-negative but contains a moderate number of CD73-positive fibrocytes within the tumour stroma, × 120.

**Figure 4** This ductal invasive carcinoma (grade II), which was oestrogen receptor negative expresses low amounts of CD73 as do the stromal fibroblasts, × 120.

was CD73-negative in 28 out of 49 cases (57%). In two out of 49 cases (4%) it was mixed, negative and weakly positive. Nineteen out of 49 cases (39%) contained strongly positive myoepithelial cells.

*Breast carcinoma*

Investigating the CD73 antigen expression of the 102 breast carcinomas, 93 out of 102 (91%) were completely negative (Figure 2), four out of 102 (4%) contained both CD73-negative and -positive tumour cells and five out of 102 (5%)

**Figure 5** This ductal invasive carcinoma (grade II–III) which was biochemically oestrogen receptor positive contains high amounts of strongly CD73-positive stromal fibroblasts but does by itself not express ecto-5′-NT, × 120.

were weakly but entirely CD73-positive (Figure 4). Distorted and/or destroyed glandular remnants within the carcinomas were occasionally observed to express CD73 (e.g., Figure 2); however, the extent of CD73-positivity of this non-neoplastic compartment could not be exactly determined since it was not sufficiently well discernible as such from the carcinoma itself. Differences in the intensity of staining of myoepithelial cells in non-cancerous breast as compared to reactive myoepithelial remnants within cancerous lesions were not obvious.

The mode of CD73 expression in fibroblasts of the tumour stroma tended to be opposite to that of neoplastic cells. Seven out of 102 carcinomas (7%) had CD73-negative fibroblasts of the tumour stroma, whereas in 26 out of 102 carcinomas (25%), the fibroblast population was mixed with small numbers of negative and a majority of strongly CD73-positive cells (Figure 3; Figure 5). In 69 out of 102 tumours (68%) the fibroblast compartment was completely and strongly CD73-positive.

Statistical analysis revealed no correlation between the mode of CD73 expression of the glandular and myoepithelial compartment in non-neoplastic breast on the one hand and age or menopausal status on the other hand. The mode of CD73 expression within the carcinoma cell compartment was neither correlated with the patients' age or menopausal status nor with the hormone receptor status, histological tumour type, grade of differentiation or postoperative staging of the tumours. However, a significant positive correlation was found between CD73 antigen expression in the stromal fibroblasts and the biochemically determined ER status of the tumours ($P < 0.038$).

### Discussion

We have shown that the normal and fibrocystic state of the non-lactating mammarian gland is characterised by a variable CD73 expression in lobular and ductal epithelium and in the myoepithelial compartment. Reactive gland which could be detected in the vicinity of half of the tumour tissues examined showed a roughly corresponding pattern of CD73-positivity: ductal and acinar epithelial cells were positive in 20% of the cases; (subsets of) myoepithelial cells contained the antigen in 43%.

As compared to their normal counterparts, i.e., lobular and ductal epithelium, carcinoma cells expressed the antigen even more infrequently: only 9% of the cases were completely CD73-positive or contained variable amounts of CD73-positive tumour cells. This might be due to a loss of the capacity to express ecto-5′-NT upon stimuli yet to be defined. In contrast to the rare expression of CD73 antigen in tumour cells, the cells of the tumour stroma were completely CD73-positive in 68% and predominantly positive in 26% of the cases. The fibroblasts of the tumour stroma were completely CD73-negative in only 7% of carcinomas.

The functional role of ecto-5′-NT (CD73) in cellular components of normal and neoplastic breast tissue has not yet been defined. Ecto-5′-NT catalyses the extracellular dephosphorylation of purine and pyrimidine ribo- and deoxyribonucleotide monophosphates to the corresponding ribo- and deoxyribo-nucleosides (Naito & Lowenstein, 1981). These compounds then may be transported inside the cell and reconverted to nucleotides via the purine salvage pathway. Thereby the enzyme can regulate the uptake of purines by converting non-transportable 5′-nucleotides (mainly adenosine monophosphates) into a transportable form (Shah *et al.*, 1986; Thompson, 1985).

In lymphocytes, it was shown that the catalytic activity of ecto-5′-NT can provide the total purine requirements of mitogen-stimulated human T cells and rapidly dividing human B lymphoblastoid cells (Thompson, 1985). Anti-5′-nucleotidase-IgG completely depressed cell proliferation, showing clearly that this is the only enzyme on the lymphocyte surface that is capable of degrading extracellular nucleotides (Andree *et al.*, 1987). Whether or not ecto-5′-NT may salvage extracellular nucleotides in breast tissue, however, is still unknown.

Ecto-5′-NT is thought to be a maturation marker for both T and B cells because of its significant higher enzyme activity in the peripheral lymphocytes than in immature precursor cells (Thompson *et al.*, 1989). Inhibition of ecto-5′-NT activity suppressed the proliferative and cytotoxic response of alloreactive T lymphocytes. These studies suggest a critical role of this cell surface enzyme in the functional maturation of both T and B lymphocytes (Massaia *et al.*, 1988). In addition, it was reported that substantial T cell proliferation can be induced when T lymphocytes are cultured for 3 days in the presence of CD73 monoclonal antibody 1E9.28.1 plus phorbol myristate acetate (PMA) and F(ab′)2 goat anti-mouse IgG as a cross-linking reagent (Thompson, 1990) which indicates a possible agonistic effect of the monoclonal antibody to its receptor. Extending this knowledge of the functional role of ecto-5′-NT in lymphocytes to breast tissue cells, we conclude that CD73 antigen expression could be a sign of their maturation state and/or susceptibility to activation.

A number of tumour-cell produced substances are known to induce or act directly on the adjacent stroma cells (Dvorak, 1986; Martinez-Hernandez, 1988). Tumours secrete substances such as tumour angiogenesis factor (Folkman, 1985), which are capable of acting on vascular structures. The extracellular matrix secreted by human breast cancer cells has been shown to be mitogenic for fibroblasts (Kao *et al.*, 1984). Transforming growth factors (TGF alpha, TGF beta) are able to cause reversible phenotypic transformation of fibroblasts (Hsuan, 1989). In hormone dependent MCF-7 breast cancer cells, oestrogen-induced growth factors were identified that may have a role in growth control and might act as oestrogen-induced ‘second messengers’ in oestrogen-responsive growth of human breast cancer (Dickson *et al.*, 1986; Knabbe *et al.*, 1987). Our statistical analysis revealed an interdependence between the oestrogen receptor status of breast carcinomas and the ecto-5′-NT expression of the adjacent stromal fibroblasts. Whether any of these effects of oestrogen-induced growth factors are mediated via CD73 will now have to be investigated.

This study was supported by a grant of the Tumorzentrum Heidelberg/Mannheim.

### References

ANDREE, T., GUTENSOHN, W. & KUMMER, U. (1987). Is ecto-5′-nucleotidase essential for stimulation of human lymphocytes? evidence against a role of the enzymes as mitogenic lectin receptor. *Immunobiol.*, **175,** 214.

BASTIAN, J.F, RUEDI, J.M., MACPHERSON, G.A., GOLEMBESKY, H.E., O'CONNOR, R.D. & THOMPSON, L.F. (1984). Lymphocyte ecto-5′-nucleotidase activity in infancy: increasing activity in peripheral blood B cells precedes their ability to synthesis IgG *in vitro*. *J. Immunol.*, **132,** 1767.

BAILYES, E.M., FERGUSON, M.A.J., COLACO, C.A.L.S. & LUZIO, J.P. (1990). Inositol is a constituent of detergent-solubilized immuno-affinity-purified rat liver 5′-nucleotidase. *Biochem. J.*, **265,** 907.

BLOOM, H.J.G. & RICHARDSON, W.W. (1957). Histologic grading and prognosis in breast cancer. A study of 1409 cases of which 359 have been followed for 15 years. *Br. J. Cancer*, **11,** 359.

BOYLE, J.M., HEY, Y., GUERTS VAN KESSEL, A. & FOX, M. (1988). Assignment of ecto-5′-nucleotidase to human chromosome 6. *Hum. Genet.*, **81,** 88.

DICKSON, R.B., MCMANAWAY, M.E. & LIPPMAN, M.E. (1986). Estrogen-induced factors of breast cancer cells partially replace estrogen to promote tumour growth. *Science*, **232,** 1540.

DVORAK, J.F. (1986). Tumours: wounds that do not heal (similarities between tumour stroma generation and wound healing). *N. Engl. J. Med.*, **315,** 1650.

EDWARDS, N.L., GELFAND, E.W., BURK, L., DOSCH, H.M. & FOX, I.H. (1979). Distribution of 5′-nucleotidase in human lymphoid tissues. *Proc. Natl Acad. Sci. USA*, **76,** 3474.

FOLKMAN, J. (1985). Tumor angiogenesis. *Adv. Cancer Res.*, **43,** 175.

HSUAN, J.J. (1989). Transforming growth factors (beta). *Br. Med. B.*, **45,** 425.

KAO, R.T., HALL, J. & STERN, R. (1986). Collagen and elastin synthesis in human stroma and breast carcinoma cell lines; modulation by the extracellular matrix. *Tissue Res.*, **14,** 245.

KNABBE, C., LIPPMANN, M.E., WAKEFIELD, L.M. & 4 others (1987). Evidence that transforming growth factor(beta) is a hormonally regulated negative growth factor in human breast cancer cells. *Cell*, **48,** 417.

KUMMER, U., MYSLIWIETZ, J., GUTENSOHN, W. & 4 others (1984). Development and properties of a monoclonal antibody specific for human ecto-5′-nucleotidase. *Immunobiol.*, **166,** 203.

LOW, M.G. (1989). Glycosyl-phosphatidylinositol: a versatile anchor for cell surface protein. *FASEB J.*, **3,** 1600.

LOW, M.G. & FINEAN, J.B. (1978). Specific release of plasma membrane enzymes by a phosphatidylinositol-specific phospholipase C. *Biochim. Biophys. Acta.*, **508,** 565.

MARTINEZ-HERNANDEZ, A. (1988). The extracellular matrix and neoplasia. *Lab. Invest.*, **58,** 609.

MASSAIA, M., PILERI, A., BOCCADORO, M. BIANCHI, A., PALUMBO, A. & DIANZANI, U. (1988). The generation of alloreactive cytotoxic T lymphocytes requires the expression of ecto-5′-nucleotidase activity. *J. Immunol.*, **141,** 3768.

MISUMI, Y., OGATA, S., HIROSE, S. & IKEHARA, Y. (1990). Primary structure of rat liver 5′-nucleotidase decuded from the cDNA. Presence of the COOH-terminal hydrophobic domain for possible post-translational modification by glycophospholipid. *J. Biol. Chem.*, **265,** 907.

MÖLLER, P. & MIELKE, B. (1989). B-cell antigens: Workshop report. Extensive analysis of tissue distribution of antigens defined by new clustered and unclustered B-cell antibodies. In: Knapp, W., Dörken, B., Rieber, P., Schmidt, R.E., Stein, H. & von dem Borne, A.E.G.Kr (eds). *Leukocyte Typing IV*. Oxford University Press, Oxford.

NAITO, Y. & LOWENSTEIN, J.M. (1981). 5′-nucleotidase from rat heart. *Biochemistry*, **20,** 5188.

RAAM, S., GELMAN, R. & FAULKNER, J. (1982). Quality control for estrogen receptor quantification by dextran-coated charcoal assay: a single laboratory's experience. *Breast Cancer Res. Treat.*, **2,** 111.

ROBINSON, P.J., MILLRAIN, M. ANTONIOU, J., SIMPSON, E. & MELLOR, A.L. (1989). A glycophospholipid anchor is required for Qa-2-mediated T cell activation. *Nature*, **342,** 85.

SHAH, T., SIMPSON, R.J., WEBSTER, A.D.B. & PETERS, T.J. (1986). Uptake of free adenosine and adenosine from adenosine monophosphate by human peripheral blood lymphocytes: possible kinetic role for ecto-5′-nucleotidase in the regulation of intracellular adenosine. *Clin. Exp. Immunol.*, **66,** 158.

THOMPSON, L.F. (1985). Ecto-5′-nucleotidase can provide the total purine requirements of mitogen-stimulated human T cells and rapidly dividing human B lymphoblastoid cells. *J. Immunol.*, **134,** 3794.

THOMPSON, L.F,. RUEDI, J.M., GLASS, A., LOW, M.G. & LUCAS, A.H. (1989). Antibodies to 5'-Nucleotidase (CD73), A glycosyl-phosphatidylinositol-anchored protein cause human peripheral blood T cells to proliferate. *J. Immunol.*, **143**, 1815.

THOMPSON, L.F., RUEDI, J.M., GLASS, A. & 6 others (1990). Production and characterization of monoclonal antibodies to the glycosyl phosphatidylinositol-anchored lymphocyte differentiation antigens ecto-5'-nucleotidase (CD73). *Tissue Antigens*, **35**, 9.

THOMPSON, L.F., RUEDI, J.M. & LOW, G. (1987). Purification of 5'-Nucleotidase from human placenta after release from plasma membranes by phosphatidylinositol-specific phospholipase C. *Biochim. Biophys. Acta*, **145**, 118.

THOMPSON, L.F., RUEDI, J.M., O'CONNOR, R.D. & BASTIAN, J.F. (1986). Ecto-5'-nucleotidase expression during human B cell development. An explanation for the heterogeneity in B lymphocyte ecto-5'nucleotidase activity in patients with hypo-gammaglobulinemia. *J. Immunol.*, **137**, 2496.

UICC: TNM CLASSIFICATION OF MALIGNANT TUMOURS (1987). 4th ed. Hermanek, P. & Sobin, L.H. (ed.). Springer: Berlin, Heidelberg, New York, London, Paris, Tokyo.

UICC: HISTOLOGICAL TYPING OF BREAST TUMOURS (1981). 2nd ed. Azzopardi, J.G. *et al.* (ed.). World Health Organization: Geneva.